# Existence of Limit Cycles in the Solow Model with Delayed-Logistic Population Growth

**DOI:** 10.1155/2014/207806

**Published:** 2014-01-28

**Authors:** Carlo Bianca, Luca Guerrini

**Affiliations:** ^1^Dipartimento di Scienze Matematiche, Politecnico, Corso Duca degli Abruzzi 24, 10129 Torino, Italy; ^2^Department of Management, Polytechnic University of Marche, 60121 Ancona, Italy

## Abstract

This paper is devoted to the existence and stability analysis of limit cycles in a delayed mathematical model for the economy growth. Specifically the Solow model is further improved by inserting the time delay into the logistic population growth rate. Moreover, by choosing the time delay as a bifurcation parameter, we prove that the system loses its stability and a Hopf bifurcation occurs when time delay passes through critical values. Finally, numerical simulations are carried out for supporting the analytical results.

## 1. Introduction

Investigations on the existence of limit cycles and the related stability analysis in nonlinear autonomous differential equations go back to Poincaré [1], who has proved that if differential equations admit a limit cycle then the difference between the number of nodes, centers, and foci enclosed by a limit cycle and the number of enclosed saddle points are equal to one. In 1924 Ivar Otto Bendixson established a sufficient condition for nonexistence of limit cycles [2]. Thereafter, the existence of limit cycles in systems of nonlinear differential equations has been of great interest in the pure and applied mathematics; see the survey [3].

This paper is concerned with the existence and stability analysis of a differential equations system, with time delay, that can be proposed for the modeling of the economic growth.

The origin of the economic growth theory goes back to Solow [4] and Swan's [5] neoclassical growth theory. A standard assumption in the economic growth theory is that population always grows at a constant and positive rate. Recently, the idea that such a specification is unrealistic has been arisen. Indeed this assumption implies, as in the Malthus model [6], that the population size goes to infinity as time goes to infinity.

Assuming a nonconstant population growth rate but variable and bounded over time, Guerrini proposed in [7] a generalization of the Solow model. Specifically a standard neoclassical technology and a Verhulst [8] logistic population growth law are assumed. The mathematical analysis performed in [7] shows the existence of a unique globally stable steady state to which the economy adjusts, so sharing the technical and qualitative properties with the Solow model.

It is well known that, in the logistic law models, the time evolution of the population density at time *t* depends on the population density at the same time. Obviously, this is a roughly approximation of the reality, since the real process of reproduction is not instantaneous in time and is strictly dependent on the previous instants of time. Therefore, the logistic law appears to be inadequate for the time description of the population density growth; the interested reader is referred to Hutchinson [9] for a complete description in the case of the ecology.

Bearing all the above in mind, the logistic law has been modified and a time delay has been inserted. The resulting mathematical model is known as the delayed logistic equation or Hutchinson's equation. It is worth precising that the introduction of time delay has been carried out also in biological systems, see paper [10], and in economics systems (see [11–15]).

The present paper aims at investigating how the delayed logistic law may affect the dynamics of the Solow model and consequently provokes the existence and the stability of limit cycles and Hopf bifurcations. The corresponding mathematical model is a system of two first-order nonlinear delayed differential equations, whose linear stability is discussed by analyzing the associated characteristic transcendental equation. The analytical results show that, as the magnitude of the time delay increases, the system loses its stability and a Hopf bifurcation occurs when the time delay passes through a sequence of critical values. Numerical simulations refer to a sensitive analysis on the time delay and support the analytical results.

It is worth stressing that the problem whether time delay may induce cyclic behavior in the Solow economic growth model has been already discussed in the pertinent literature; see, among others, paper [16] and the references cited therein.

The contents of this paper are outlined as follows. After this introduction, Section 2 is meant to be the description of the economy where the delayed Solow model is derived. Analytical investigations on the existence of steady states, stability and Hopf bifurcation analysis are dealt with in Section 3.  Section 4 is concerned with further analysis on the Hopf bifurcation, namely, the direction of the bifurcation (when it is subcritical or supercritical) and the bifurcating period. Numerical simulations obtained by sensitive analysis on the magnitude of the time delay are performed in Section 5. A critical analysis of the results and discussions of future research perspectives are postponed to Section 6.

## 2. The Delayed Mathematical Model

This section deals with the derivation of the Solow mathematical model with a time delay. Specifically a closed economy is considered, which consists of a single good *Y* = *Y*(*t*) that is used either for consumption or investment. The good is produced by labor *L* = *L*(*t*) and physical capital *K* = *K*(*t*) accordingly to a neoclassical production function *Y* = *F*(*K*, *L*); see [17]. Each worker has a unit of time available each period that is supplied inelastically in the labor market. There is a full employment in the economy, so that population size and workforce can be used interchangeably. Since the economy is assumed to be closed, change in the capital stock equals gross investment less depreciation, namely,

(1) $$\begin{array}{c} \overset{.}{K}=sY-\delta K, \end{array}$$

where *s* denotes the constant fraction of the gross income *Y* saved and *δ* is the constant fraction of capital stock that disappears as a result of depreciation. Population growth rate evolves according to the following delayed logistic equation [9]:

(2) $$\begin{array}{c} \overset{.}{L}=L\left(a-bL_{d}\right), \end{array}$$

where *L*_*d*_ : = *L*(*t* − *τ*), *τ* > 0 is the time delay, and *a* > *b* > 0 are positive constants such that *a* − *bL*(0) > 0, with *L*(0) being the density of the population at the initial time. Setting *k* = *K*/*L* and writing the production function in intensive form *f*(*k*) = *F*(*K*/*L*, 1), we obtain the following mathematical model described by two nonlinear delayed differential equations:

(3) k.=sf(k)−(δ+a−bLd)k,L.=L(a−bLd),

for some initial function *L*(*t*) = *φ*(*t*), for *t* ∈ [−*τ*, 0]. In contrast to classical dynamical systems with zero delay, the definition of the initial function *φ*, usually called history function, is required. The domain of *φ* is defined over the range of time delimited by the time delay.

It is worth stressing that with respect to the paper [18], the mathematical model considered in this paper (to which we refer as the Solow model with delayed-logistic population rate) includes the time delay in the logistic term. Moreover, the mathematical analysis will be performed with respect to an arbitrary neoclassical production function *f*.

## 3. Asymptotic Analysis: Steady States and Hopf Bifurcation

This section is devoted to the study of the asymptotic behavior of the mathematical model (3). The main goal of the present paper is to investigate the existence of equilibrium points (steady states), their stability analysis, and the possible existence of Hopf bifurcations.

The steady states of the mathematical model (3) coincide with the steady states of the corresponding model with zero delay. Hence, there exists a unique nontrivial steady state (*k*_∗_, *L*_∗_), such that *sf*(*k*_∗_) = *δk*_∗_ and *L*_∗_ = *a*/*b*. Let *x* = *k* − *k*_∗_ and *y* = *L* − *L*_∗_, so that the equilibrium in (3) is shifted to the origin. By applying in system (3) the Taylor expansion with center the origin, the model (3) recasts into the following system:

(4) $$\begin{array}{c} \overset{.}{x}=\left[sf^{\prime }\left(k_{*}\right)-\delta \right]x+bk_{*}y_{d}+g_{1}\left(x,y_{d}\right),\\ \overset{.}{y}=-ay_{d}+g_{2}\left(y,y_{d}\right), \end{array}$$

where the nonlinear parts *g*_1_(*x*, *y*_*d*_), *g*_2_(*y*, *y*_*d*_) read:

(5) g1(x,yd)=12[sf′′(k∗)x2+2bxyd]+13![sf′′′(k∗)x3]+⋯,g2(y,yd)=12(−b)yyd,

where *y*_*d*_ = *y*(*t* − *τ*). Writing the linear part of system (4) in matrix form

(6) $$\begin{array}{c} \left[\begin{array}{c} \overset{.}{x}\\ \overset{.}{y} \end{array}\right]=\left[\begin{array}{cc} sf^{\prime }\left(k_{*}\right)-\delta & 0\\ 0 & 0 \end{array}\right]\left[\begin{array}{c} x\\ y \end{array}\right]+\left[\begin{array}{cc} 0 & bk_{*}\\ 0 & -a \end{array}\right]\left[\begin{array}{c} x_{d}\\ y_{d} \end{array}\right], \end{array}$$

it is immediately seen that the resulting characteristic equation is

(7) |sf′(k∗)−δ−λbk∗e−λτ0−λ−ae−λτ|  =[sf′(k∗)−δ−λ](−λ−ae−λτ)=0.

Equation (7) is a quasi-polynomial equation, which has, in general, an infinite number of (complex) roots. When there is no time delay, that is, *τ* = 0, the characteristic equation reduces to a simple quadratic equation with two negative real characteristic roots, *λ* = −*a* and *λ* = *sf*′(*k*_∗_) − *δ* = −*s*[*f*(*k*_∗_) − *k*_∗_ *f*′(*k*_∗_)]/*k*_∗_ < 0. Thus, the steady state of system (4) is locally asymptotically stable.

Let *τ* > 0 be fixed. Taking the time delay as a bifurcation parameter, we investigate the effects of the time delay on the dynamics of the model (3). Accordingly, we look for values of *τ* so that the steady state changes its asymptotic stability from local stability to instability.

It is well known that the steady state of system (4) is locally asymptotically stable if each of the characteristic roots of (7) has negative real part. Since *λ* = 0 is not a zero of (7), we need to examine only when this equation has pure imaginary roots *λ* = ±*iω*, where *ω* is a positive real number.

**Lemma 1**

The characteristic equation (7) has a pair of simple conjugate pure imaginary roots *λ* = ±*iω*_0_ (*ω*_0_ > 0) at *τ* = *τ*_*j*_, where

(8) $$\begin{array}{c} \omega_{0}=a,\;\;\tau_{j}=\frac{1}{\omega_{0}}\left(\frac{\pi }{2}+2j\pi \right),\;j=0,1,2,\ldots . \end{array}$$

Furthermore, the following transversality condition:

(9) d[Reλ(τ)]dτ|τ=τj>0

holds true.

**Proof**

Let *λ* = *iω*  (*ω* > 0) be a root of the characteristic equation (7). Then *λ* must be a root of

(10) $$\begin{array}{c} D\left(\lambda ,\tau \right)=-\lambda -ae^{-\lambda \tau }=0. \end{array}$$

Substituting *λ* = *iω* into (10), we have

(11) $$\begin{array}{c} i\omega +a\left(\cos\omega \tau -i\sin\omega \tau \right)=0. \end{array}$$

Separating the real and imaginary parts yields

(12) $$\begin{array}{c} \omega =a\sin\omega \tau ,\;\;\cos\omega \tau =0. \end{array}$$

Since *ω* and *a* are positive constants, *ωτ* = *π*/2 + 2*jπ*, *j* = 0,1, 2,…, and *ω* = *a* = *ω*_0_. To ensure that the roots *λ* = ±*iω*_0_ are simple roots, we differentiate (10) with respect to *λ* and we obtain *dD*(*λ*, *τ*)/*dλ* = −1 + *aτe* ^−*λτ*^. It is easy to see that the conditions

(13) $$\begin{array}{c} D\left(\pm i\omega_{0},\tau \right)=0,\;\;\frac{dD\left(\pm i\omega_{0},\tau \right)}{d\lambda }=0, \end{array}$$

hold true only if −1 ± *iω*_0_ *τ* = 0. This concludes the first part of the statement.

Next, let *λ*(*τ*) = *μ*(*τ*) + *iω*(*τ*) be the root of (7) such that *μ*(*τ*_*j*_) = 0 and *ω*(*τ*_*j*_) = *ω*_0_. Differentiating (10) with respect to *τ* and using (10), one has

(14) $$\begin{array}{c} \left(1+\lambda \tau \right)\frac{d\lambda }{d\tau }=-\lambda^{2}. \end{array}$$

On the other hand,

(15) sign⁡{d[Reλ(τ)]dτ|τ=τj}=sign⁡{Re(dλdτ)−1|τ=τj}=sign⁡{Re(−1λ2−τλ)|τ=τj}=sign⁡{1ω02}.

Then the proof of the theorem has been gained.

Since the sign of *d*[*Reλ*(*τ*_*j*_)]/*dτ* is positive, then each crossing of the real part of the characteristic roots at *τ*_*j*_ must be from left to right. With being the crossing direction, always toward instability, the following result on the distribution of roots of (7) is obtained.

**Proposition 2**

If *τ* ∈ [0, *τ*_0_), all roots of (7) have negative real parts. If *τ* = *τ*_0_, all roots of (7), except for ±*iω*_0_, have negative real parts. If *τ* ∈ (*τ*_*j*_, *τ*_*j*+1_), for *j* = 0,1, 2,…, (7) has 2(*j* + 1) roots with positive real parts.

Bearing all the above in mind, we have that the hypotheses for Hopf bifurcation are satisfied at *τ*_*j*_. These allow us to prove the following theorem.

**Theorem 3**

The positive steady state (*k*_∗_, *L*_∗_) of system (3) is locally asymptotically stable for *τ* ∈ [0, *τ*_0_) and unstable for *τ* > *τ*_0_. Moreover, system (3) undergoes a Hopf bifurcation at (*k*_∗_, *L*_∗_), when *τ* = *τ*_*j*_, for *j* = 0,1, 2,….

**Proof**

Spectral properties stated in Proposition 2 lead immediately to stability properties of the zero equilibrium of system (4) and of the positive equilibrium (*k*_∗_, *L*_∗_) of system (3). Hence, we have the proof.

## 4. Analysis of the Hopf Bifurcations

This section is meant to the study of the direction of the Hopf bifurcation and the stability of bifurcating periodic solutions. The results are obtained by applying the normal form theory and the center manifold theorem stated in [19].

As already shown, the system (3) undergoes a Hopf bifurcation at the positive equilibrium point (*k*_∗_, *L*_∗_) when *τ* = *τ*_*j*_ and *λ* = *iω*_0_ is the corresponding purely imaginary root of the characteristic equation at this point. Setting *τ* = *τ*_*j*_ + *μ*, *μ* ∈ ℝ, then *μ* = 0 is the Hopf bifurcation value of system (3) for the new bifurcation parameter *μ*.

Let *C* = *C*([−*τ*_*j*_, 0], ℝ^2^) be the space of continuous real-valued functions. For any *φ* = (*φ*_1_, *φ*_2_) ∈ *C*, let

(16) Lμ(φ)=[sf′(k∗)−δ000][φ1(0)φ2(0)]+[0bk∗0−a][φ1(−τj)φ2(−τj)],

(17) $$\begin{array}{c} g\left(\tau_{j},\varphi \right)=\left[\begin{array}{c} g_{1}\left(\tau_{j},\varphi \right)\\ g_{2}\left(\tau_{j},\varphi \right) \end{array}\right], \end{array}$$

where

(18) g1(τj,φ)=12[sf′′(k∗)φ1(0)2+2bφ1(0)φ2(−τj)]+13![sf′′′(k∗)φ1(0)3]+⋯,g2(τj,φ)=12(−b)φ2(0)φ2(−τj).

By Riesz's representation theorem, there exists a matrix whose components are bounded variation functions *η*(*θ*, *μ*) in *θ* ∈ [−*τ*_*j*_, 0] such that

(19) $$\begin{array}{c} L_{\mu }\varphi =\overset{0}{\underset{-\tau_{j}}{\int }}\left[d\eta \left(\theta ,\mu \right)\right]\varphi \left(\theta \right),\;\text{for}\;\;\varphi \in C. \end{array}$$

In fact, we can choose

(20) $$\begin{array}{c} \eta \left(\theta ,\mu \right)=\left[\begin{array}{cc} sf^{\prime }\left(k_{*}\right)-\delta & 0\\ 0 & 0 \end{array}\right]\Gamma \left(\theta \right)+\left[\begin{array}{cc} 0 & bk_{*}\\ 0 & -a \end{array}\right]\Gamma \left(\theta +\tau_{j}\right), \end{array}$$

where Γ(*θ*) is the Dirac delta function. For *φ* ∈ *C*, we define

(21) $$\begin{array}{c} A\left(\mu \right)\left(\varphi \right)=\{\begin{array}{cc} \frac{d\varphi \left(\theta \right)}{d\theta }, & \theta \in \left[-\tau_{j},0\right),\\ \overset{0}{\underset{-\tau_{j}}{\int }}\left[d\eta \left(r,\mu \right)\right]\varphi \left(r\right)‍, & \theta =0, \end{array}\\ R\left(\mu \right)\left(\varphi \right)=\{\begin{array}{cc} 0, & \theta \in \left[-\tau_{j},0\right),\\ g\left(\mu ,\varphi \right), & \theta =0. \end{array} \end{array}$$

Then system (4) can be rewritten in the following form:

(22) $$\begin{array}{c} {\overset{.}{u}}_{t}=A\left(\mu \right)u_{t}+R\left(\mu \right)u_{t}, \end{array}$$

where *u*_*t*_ = *u*(*t* + *θ*), for *θ* ∈ [−*τ*_*j*_, 0]. For $\psi \in \tilde{C}=C([0,\tau_{j}],{\mathbb{R}}^{2})$, we define

(23) $$\begin{array}{c} A^{*}\left(\mu \right)\psi \left(r\right)=\{\begin{array}{cc} -\frac{d\psi \left(r\right)}{dr}, & r\in \left(0,\tau_{j}\right],\\ \overset{0}{\underset{-\tau_{j}}{\int }}d\eta \left(\zeta ,\mu \right)\psi \left(-\zeta \right)‍, & r=0. \end{array} \end{array}$$

For *φ* ∈ *C* and $\psi \in \tilde{C}$, we define the following bilinear form:

(24) 〈ψ(r),φ(θ)〉=ψ−(0)φ(0)−∫θ=−τj0∫ξ=0θψ−(ξ−θ)dη(θ,0)φ(ξ)dξ‍‍.

Therefore, *A*(0) and A*(0) are adjoint operators. Let *q*(*θ*) and q*(*r*) be the eigenvectors of *A*(0) and A*(0) corresponding to the eigenvalues *iω*_0_ and −*iω*_0_, respectively, and normalized so that 〈q*(*r*), *q*(*θ*)〉 = 1. It is well known that *q*(*θ*) can be computed from *A*(0)*q*(*θ*) = *iω*_0_ *q*(*θ*), where *q*(*θ*) = *q*(0)*e* ^*iω*_0_*θ*^, similarly for q*(*r*). For a solution *u*_*t*_ of (17) at *μ* = 0, we define

(25) z(t)=〈q∗,ut〉,W(t,θ)=ut(θ)−2Re[z(t)q(θ)],

where

(26) W(t,θ)=W(z(t),z−(t),θ)=W20(θ)z22+W11(θ)zz−+W02(θ)z−22+⋯.

Then (17) valued in the points of the center manifold *𝒞* is described by

(27) z.(t)=〈q∗,z.t〉=〈q∗,A(0)ut+R(0)ut〉:=iω0z+q−∗(0)g0,

where *g*_0_ = *g*(0, *u*_*t*_), with *g* defined by (17). Now (26) can be written as

(28) $$\begin{array}{c} \overset{.}{z}\left(t\right)=i\omega_{0}z+g\left(z,\overset{-}{z}\right), \end{array}$$

with

(29) $$\begin{array}{c} g\left(z,\overset{-}{z}\right)=g_{20}\frac{z^{2}}{2}+g_{11}z\overset{-}{z}+g_{02}\frac{{\overset{-}{z}}^{2}}{2}+g_{21}\frac{z^{2}\overset{-}{z}}{2}+\cdots . \end{array}$$

Next, substitute *u*_*t*_(*θ*) = *W*(*t*, *θ*) + 2*Re*[*z*(*t*)*q*(*θ*)] into *g*_0_ and denote

(30) $$\begin{array}{c} g_{0}=g_{z^{2}}\frac{z^{2}}{2}+g_{z\overset{-}{z}}z\overset{-}{z}+g_{{\overset{-}{z}}^{2}}\frac{{\overset{-}{z}}^{2}}{2}+g_{z^{2}\overset{-}{z}}\frac{z^{2}\overset{-}{z}}{2}+\cdots . \end{array}$$

Hence, from $g(z,\overset{-}{z})={\overset{-}{q}}^{*}(0)g_{0}$, we obtain

(31) $$\begin{array}{c} g_{20}={\overset{-}{q}}^{*}\left(0\right)g_{z^{2}},\\ g_{02}={\overset{-}{q}}^{*}\left(0\right)g_{{\overset{-}{z}}^{2}},\\ g_{11}={\overset{-}{q}}^{*}\left(0\right)g_{z\overset{-}{z}},\\ g_{21}={\overset{-}{q}}^{*}\left(0\right)g_{z^{2}\overset{-}{z}}. \end{array}$$

The next step is to compute *W*_20_(*θ*) and *W*_11_(*θ*) since the term *g*_21_ is dependent on them. By (22) and (28) we have

(32) W.=u.t−z.q−z−.q−={A(0)W−2Re[q−∗(0)g0q(θ)],θ∈[−τj,0),A(0)W−2Re[q−∗(0)g0q(0),]+g0,θ=0:=A(0)W+H(z,z−,θ),

where

(33) $$\begin{array}{c} H\left(z,\overset{-}{z},\theta \right)=H_{20}\left(\theta \right)\frac{z^{2}}{2}+H_{11}\left(\theta \right)z\overset{-}{z}+H_{02}\left(\theta \right)\frac{{\overset{-}{z}}^{2}}{2}+\cdots . \end{array}$$

Substituting (26) and (28) into $\overset{.}{W}=W_{z}\overset{.}{z}+W_{\overset{-}{z}}\overset{.}{\overset{-}{z}}$ and comparing the coefficients of the resulting equation with those of (32), we get

(34) $$\begin{array}{c} \left[A\left(0\right)-2i\omega_{0}\right]W_{20}\left(\theta \right)=-H_{20}\left(\theta \right),\\ A\left(0\right)W_{11}\left(\theta \right)=-H_{11}\left(\theta \right),\\ \left[A\left(0\right)+2i\omega_{0}\right]W_{02}\left(\theta \right)=-H_{02}\left(\theta \right). \end{array}$$

It follows that

(35) $$\begin{array}{c} {\overset{.}{W}}_{20}\left(\theta \right)=2i\omega_{0}W_{20}\left(\theta \right)-H_{20}\left(\theta \right),\\ {\overset{.}{W}}_{11}\left(\theta \right)=-H_{11}\left(\theta \right). \end{array}$$

Comparing the coefficients of (33) with (32), for *θ* ∈ [−*τ*_*j*_, 0), we obtain

(36) $$\begin{array}{c} H_{20}\left(\theta \right)=-g_{20}q\left(\theta \right)-{\overset{-}{g}}_{02}\overset{-}{q}\left(\theta \right),\\ H_{11}\left(\theta \right)=-g_{11}q\left(\theta \right)-{\overset{-}{g}}_{11}\overset{-}{q}\left(\theta \right), \end{array}$$

and for *θ* = 0 we have

(37) $$\begin{array}{c} H_{20}\left(0\right)=-g_{20}q\left(0\right)-{\overset{-}{g}}_{02}\overset{-}{q}\left(0\right)+g_{z^{2}},\\ H_{11}\left(0\right)=-g_{11}q\left(0\right)-{\overset{-}{g}}_{11}\overset{-}{q}\left(0\right)+g_{z\overset{-}{z}}. \end{array}$$

Solving (35) for *W*_20_(*θ*) and *W*_11_(*θ*), one has

(38) $$\begin{array}{c} W_{20}\left(\theta \right)=-\frac{g_{20}}{i\omega_{0}}q\left(0\right)e^{i\omega_{0}\theta }-\frac{{\overset{-}{g}}_{02}}{3i\omega_{0}}\overset{-}{q}\left(0\right)e^{-i\omega_{0}\theta }+E_{1}e^{2i\omega_{0}\theta },\\ W_{11}\left(\theta \right)=\frac{g_{11}}{i\omega_{0}}q\left(0\right)e^{i\omega_{0}\theta }-\frac{{\overset{-}{g}}_{11}}{i\omega_{0}}\overset{-}{q}\left(0\right)e^{-i\omega_{0}\theta }+E_{2}, \end{array}$$

where *E*_1_, *E*_2_ ∈ ℝ^2^ can be determined by setting *θ* = 0 in $H(z,\overset{-}{z},\theta )$. Substituting (38) into (34), using (37), and noticing that

(39) $$\begin{array}{c} \left[i\omega_{0}-\overset{0}{\underset{-\tau_{j}}{\int }}e^{i\omega_{0}\theta }d\eta \left(\theta ,0\right)‍\right]q\left(0\right)=0,\\ \left[-i\omega_{0}-\overset{0}{\underset{-\tau_{j}}{\int }}e^{-i\omega_{0}\theta }d\eta \left(\theta ,0\right)‍\right]\overset{-}{q}\left(0\right)=0, \end{array}$$

we arrive at

(40) $$\begin{array}{c} \left[2i\omega_{0}-\overset{0}{\underset{-\tau_{j}}{\int }}e^{2i\omega_{0}\theta }d\eta \left(\theta ,0\right)‍\right]E_{1}=g_{z^{2}},\\ \left[\overset{0}{\underset{-\tau_{j}}{\int }}d\eta \left(\theta ,0\right)‍\right]E_{2}=g_{z\overset{-}{z}}. \end{array}$$

As a result, *E*_1_ and *E*_2_ are calculated. Based on the foregoing analysis, we can see that each *g*_*ij*_ is determined. Therefore, we can compute the following quantities:

(41) c1(0)=i2ω0[g11g20−2|g11|2−|g02|23]+g212,μ2=−Re{c1(0)}Re{λ′(τj)},β2=2Re{c1(0)},T2=−Im⁡{c1(0)}+μ2Im⁡{λ′(τj)}ω0,

which give the properties of bifurcating periodic solutions.

**Theorem 4**

Let (*k*_∗_, *L*_∗_) be the steady state of the model (3). Then one has the following.

1. *The direction of the Hopf bifurcation of the system (3), at the equilibrium *(*k*_∗_, *L*_∗_)* for τ* = *τ*_*j*_ *, is subcritical (resp., supercritical) if μ*_2_ < 0* (resp., μ*_2_ > 0*).*
2. *The bifurcating periodic solution on the center manifold is unstable (resp., locally asymptotically stable) if β*_2_ > 0* (resp., β*_2_ < 0*).*
3. *The period of the bifurcating periodic solution decreases (resp., increases) if* *T*_2_ < 0* (resp., T*_2_ > 0*).*

## 5. Numerical Simulations

This section is concerned with some numerical simulations of the mathematical model (3). Specifically we depict the behavior of the solutions when the time delay is varied, thus performing a sensitive analysis on the parameter *τ*.

The mathematical model (3) is characterized by six nonnegative parameters which have an economic meaning and a function *f*. The set of simulations of this section is obtained by choosing *f* as the Cobb-Douglas function, namely,

(42) $$\begin{array}{c} f\left(k\right)={\left[k\left(t\right)\right]}^{\alpha }, \end{array}$$

fixing the following magnitude of the following five parameters:

(43) $$\begin{array}{c} \alpha =0.8,\;\;s=0.5,\;\;a=0.2,\\ b=0.5,\;\;\delta =0.9 \end{array}$$

and performing a sensitive analysis on the time delay *τ*.

**Figure 1:**
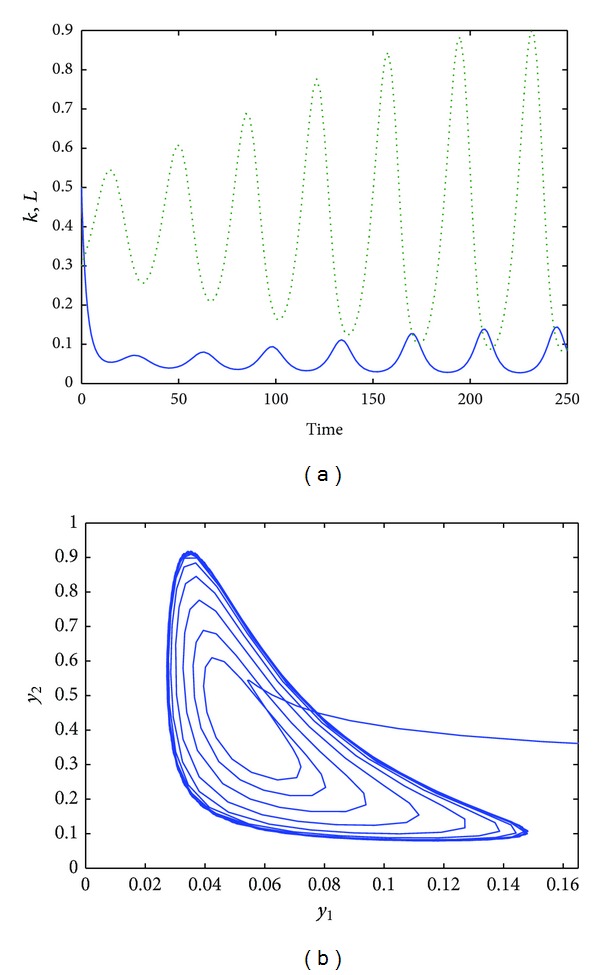
The time evolution of the functions *k*(*t*) (dotted line) and *L*(*t*) for *α* = 0.8, *s* = 0.5, *a* = 0.2, *b* = 0.5, *δ* = 0.9, and *τ* = 9 (a). The phase space of *L*(*t*) versus *k*(*t*) for *t* ∈ [0,500] (b).

**Figure 2:**
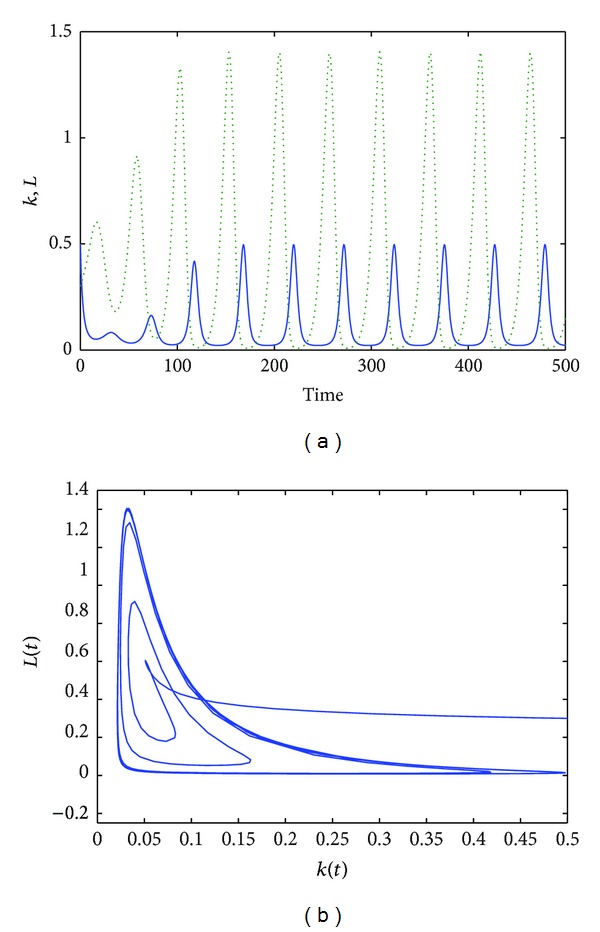
The time evolution of the functions *k*(*t*) (dotted line) and *L*(*t*) for *α* = 0.8, *s* = 0.5, *a* = 0.2, *b* = 0.5, *δ* = 0.9, and *τ* = 11 (a).

The first set of simulations refer to *τ* = 9. As Figure 1 shows, oscillations of the solutions occur (Figure 1(a)) for the all length of the simulation. A cycle limit is reached; see Figure 1(b). Further numerical simulations show that, when we increase the time delay, namely, for *τ* ≥ 9, we have a cycle limit whose period increases; see Figure 2(b). Moreover, looking at Figure 2(a), we can see that also the magnitude of the oscillations is increased.

**Figure 3:**
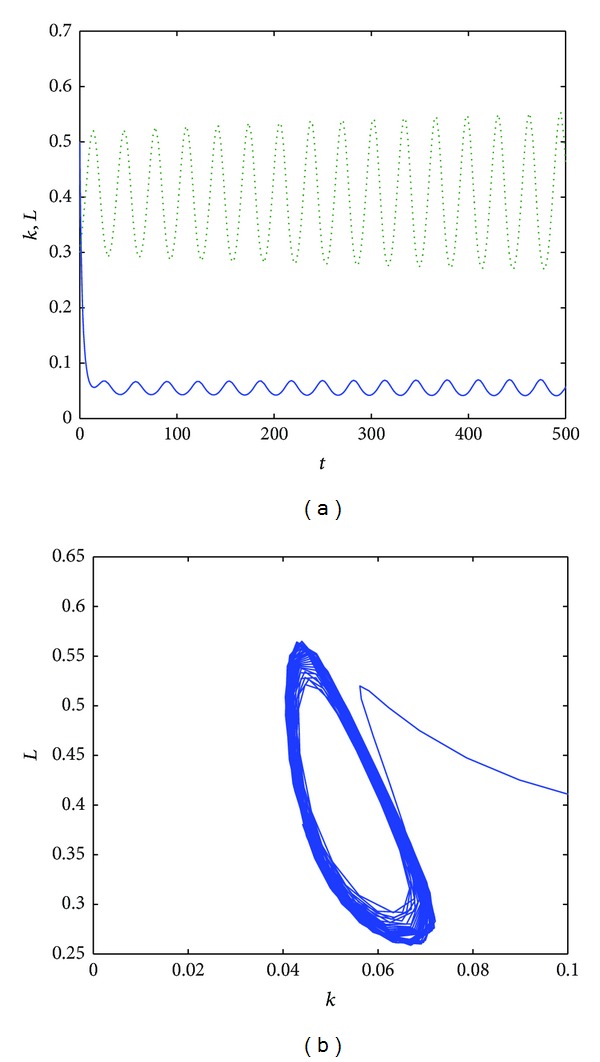
The time evolution of the functions *k*(*t*) (dotted line) and *L*(*t*) for *α* = 0.8, *s* = 0.5, *a* = 0.2, *b* = 0.5, *δ* = 0.9, and *τ* = 8 (a). The phase space of *L*(*t*) versus *k*(*t*) for *t* ∈ [0,1000] (b).

**Figure 4:**
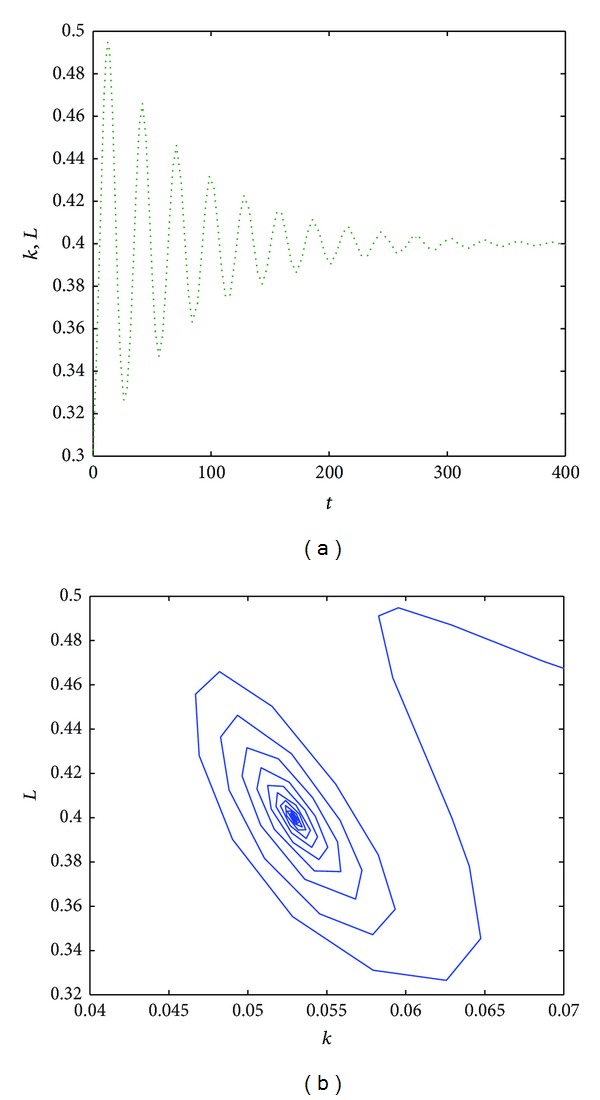
The time evolution of the functions *k*(*t*) (b) for *α* = 0.8, *s* = 0.5, *a* = 0.2, *b* = 0.5, *δ* = 0.9, and *τ* = 7 (a). The phase space of *L*(*t*) versus *k*(*t*) for *t* ∈ [0,500] (b).

When the magnitude of the time delay decreases, also the oscillations and the cycle limit period decrease; see Figure 3. Finally, when the magnitude of the time delay is less than 8, then the oscillations decrease in time and the dimension of the cycle limit is reduced; see Figure 4.

It is worth stressing that if the time delay is less than 5 then the cycle limit reduces to the point (0.05,0.4) and oscillations disappear for *τ* ≤ 2.

The above set of simulations support also the direction of the Hopf bifurcation. Indeed, a supercritical Hopf bifurcation leads from a decaying oscillation to growth and saturation of a sustained oscillation.

## 6. Conclusions and Research Perspectives

The mathematical model proposed in this paper is a generalization of the classical Solow model. Specifically the population growth rate is not assumed to be constant and a time delay is inserted in order to better approximate the real-world economy. Analytical results have shown the existence of a steady state and the possibility of a Hopf bifurcation. Moreover, the existence of limit cycles has been proved and numerical simulations, obtained by performing a sensitive analysis on the time delay parameter, have plotted the limit cycles in some cases.

The existence of limit cycles is an important topic in the economic systems. Indeed, limit cycle is a trajectory for which the economy of the system would be constant over a cycle; namely, on an average there is no loss or gain of economy. Limit cycle is an outcome of delicate energy balance due to the presence of nonlinear term into the mathematical model. Therefore, the cycles can be interpreted as economic fluctuations.

The mathematical model (3) can be further generalized by including the time delay also into the *k* function. Moreover, the time evolution of the population density can be modeled by a kinetic approach; see the review paper [20] and the references cited therein. The kinetic approach proposed in papers [21–24] allows taking into account the interactions that occur among the individuals of a population. Moreover, these models, even if they refer to nonequilibrium systems (namely, systems subjected to external force fields), can attain a nonequilibrium stationary state [25] and a bifurcation analysis can be performed. Finally, by asymptotic methods we can derive macroscopic evolution equations (see, among others, papers [26, 27]) that are of great interest in the pure [28] and applied mathematics.

It is worth mentioning that an important research perspective is the comparison of the generalized model introduced in the present paper with the experimentally measurable quantities. This is a work in progress and results will be presented to due course.
